# A Novel Mobile App (Heali) for Disease Treatment in Participants With Irritable Bowel Syndrome: Randomized Controlled Pilot Trial

**DOI:** 10.2196/24134

**Published:** 2021-03-02

**Authors:** Aaron J Rafferty, Rick Hall, Carol S Johnston

**Affiliations:** 1 College of Health Solutions Arizona State University Phoenix, AZ United States; 2 Edson College of Nursing and Health Innovation Arizona State University Phoenix, AZ United States

**Keywords:** irritable bowel syndrome, artificial intelligence, mobile app, low FODMAP diet, randomized controlled trial

## Abstract

**Background:**

A diet high in fermentable, oligo-, di-, monosaccharides and polyols (FODMAPs) has been shown to exacerbate symptoms of irritable bowel syndrome (IBS). Previous literature reports significant improvement in IBS symptoms with initiation of a low FODMAP diet (LFD) and monitored reintroduction. However, dietary adherence to the LFD is difficult, with patients stating that the information given by health care providers is often generalized and nonspecific, requiring them to search for supplementary information to fit their needs.

**Objective:**

The aim of our study was to determine whether Heali, a novel artificial intelligence dietary mobile app can improve adherence to the LFD, IBS symptom severity, and quality of life outcomes in adults with IBS or IBS-like symptoms over a 4-week period.

**Methods:**

Participants were randomized into 2 groups: the control group (CON), in which participants received educational materials, and the experimental group (APP), in which participants received access to the mobile app and educational materials. Over the course of this unblinded online trial, all participants completed a battery of 5 questionnaires at baseline and at the end of the trial to document IBS symptoms, quality of life, LFD knowledge, and LFD adherence.

**Results:**

We enrolled 58 participants in the study (29 in each group), and 25 participants completed the study in its entirety (11 and 14 for the CON and APP groups, respectively). Final, per-protocol analyses showed greater improvement in quality of life score for the APP group compared to the CON group (31.1 and 11.8, respectively; *P*=.04). Reduction in total IBS symptom severity score was 24% greater for the APP group versus the CON group. Although this did not achieve significance (–170 vs –138 respectively; *P*=.37), the reduction in the subscore for bowel habit dissatisfaction was 2-fold greater for the APP group than for the CON group (*P*=.05).

**Conclusions:**

This initial study provides preliminary evidence that Heali may provide therapeutic benefit to its users, specifically improvements in quality of life and bowel habits. Although this study was underpowered, findings from this study warrant further research in a larger sample of participants to test the efficacy of Heali app use to improve outcomes for patients with IBS.

**Trial Registration:**

ClinicalTrials.gov NCT04256551; https://clinicaltrials.gov/ct2/show/NCT04256551

## Introduction

Irritable bowel syndrome (IBS) is among the most prevalent functional gastrointestinal (GI) disorders and is characterized by a number of symptoms, including recurrent abdominal pain, altered bowel habits, bloating, and distention [1]. IBS affects roughly 15 million people within the United States [2,3] and results in yearly direct health care costs between US $30 and $75 billion [4,5], while indirect costs are estimated at an additional US $20 billion a year. Individuals with IBS often experience psychological distress and anxiety from a lack of clear understanding of the condition, being told that symptoms are all in their heads, and feelings of not being heard, all of which further exacerbate symptoms and affect quality of life [1]. Additionally, affected individuals spend significant time seeking medical support and undergoing numerous assortments of tests that all lead to a loss of work, disability, lack of productivity, and increased mortality [4,6]. Furthermore, both central factors (eg, psychological, cognitive and neuro-hormonal) and peripheral factors (eg, gut flora, genetics, and diet) have been shown to exacerbate the severity of symptoms over time [7,8].

A diet high in fermentable, oligo-, di-, monosaccharides and polyols (FODMAPs) has been shown to exacerbate symptoms of IBS [9], and research suggests adherence to a low FODMAP diet (LFD) can improve IBS symptoms [10-19]. For this reason, an LFD has become a popular tool to manage IBS and IBS-like symptoms with comparable success rates to pharmacological methods [20]. However, adherence to an LFD is difficult as FODMAP compounds are present in a variety of fruits, vegetables, grains, dairy, meats, and condiments. Moreover, patients following an LFD state that information provided by medical practitioners is often generalized and nonspecific, requiring them to search for supplementary information to fit their individual needs [21]. Support from a multidisciplinary team has been shown to mitigate these barriers to treatment of functional bowel disorders [22]; however, accessibility becomes a concern when additional barriers arise for the patient, including the time and finances needed to accommodate additional support.

Mobile apps using artificial intelligence (AI) in consort with a multidisciplinary team within a platform are gaining traction as useful tools for supporting the management of chronic conditions like diabetes and hypertension [23-26]. However, an app designed to treat IBS symptoms using AI has yet to be explored. Of the apps developed for IBS treatment, the Constant-Care web app [27], developed by researchers in Denmark, has been used by participants to successfully monitor their IBS symptoms in dietary treatment studies [10-12,27,28]. However, this app was not offered as a mobile app and only provided symptom tracking and monitoring approaches without real-time assistance for patients to improve their LFD adherence. Instead, improvement in LFD adherence relied on in-person and online education modules that could not be used in real time to make decisions [27]. To date, an app using AI to reduce IBS symptoms and improve adherence to the LFD has not been tested.

Heali AI is a personalized nutrition software company, founded in 2018 in Los Angeles, California, by a team of software engineers and registered dietitians. The Heali app uses AI to scan menus and barcodes to provide nutrition information and recommendations in accordance with user-specific individualized diet plans. Typically, app users are matched to foods based on their selected dietary preferences using a traffic light system: green (foods that fit the diet plan), yellow (foods that can be consumed in moderation), and red (foods to avoid completely). The app can be programmed to focus on a wide range of dietary preferences, including micro- and macronutrient preferences, as well as specific preprogrammed evidence-based diets such as the LFD, vegan diet, gluten-free diet, or keto diet. Once programmed, the app then helps users find foods that align with their personalized dietary needs while eating out, at the grocery store, or at home in an effort to improve dietary adherence, symptom control, and quality of life.

The purpose of this per-protocol study was to determine whether Heali, a novel AI dietary app, reduces IBS symptoms through improving adherence to the LFD as compared to standard online dietary education in populations with IBS or IBS-like symptoms after 30 days of use. It was hypothesized that, compared to standard online education, the novel AI dietary app would improve the primary outcomes of IBS symptom severity and quality of life via improving the secondary outcomes, which include adherence to an LFD and dietary knowledge related to the LFD.

## Methods

### Participants

Participants were eligible for the study if they had moderate to severe IBS based on an IBS symptom severity scale (IBS-SSS) score of 175 or greater, met Rome IV criteria for IBS, and had IBS-like symptoms for the past 3 months or longer [29]. Diagnosis using the Rome IV criteria also classifies patients by symptomology: IBS with constipation (IBS-C), IBS with diarrhea (IBS-D), IBS mixed typed (IBS-M), or IBS unsubtyped (IBS-U).

Eligibility also required that participants be between 18 and 65 years of age and own a cell phone. Exclusion criteria included use of a dietary app or elimination diet (eg, LFD, specific carbohydrate, vegan, vegetarian, gluten free, dairy free) within the last 6 months, food allergies (not including food intolerances), smoking habit, history of chronic disease other than GI dysfunction (eg, diabetes, cardiovascular disease, hypertension, eating disorders, or diseases of the lungs, kidney, liver, or thyroid), or being a nutrition student or professional.

The study was advertised via online flyers, social media posts (Facebook, Twitter, Instagram), list servers, and recruitment services across a university community. The recruitment message indicated that participants must be willing to participate in an LFD intervention over a 30-day period with a 10-day pretrial monitoring period. The message also stated that participants would be instructed to complete online questionnaires on a biweekly basis from the start of the study. Participants provided consent via email, and the study was approved by the Arizona State University Institutional Review Board. The study was registered at ClinicalTrials.gov (NCT04256551).

A total of 58 participants were recruited. According to previous literature, 20 participants per group would provide 80% power to detect at least an 81-point decrease in IBS-SSS symptom scores between groups at an α value of .05 [10,16,18,30]. Thus, assuming a retention rate of 80%, a minimum of 25 participants per group was desired.

### Study Design

This 40-day randomized controlled experimental study consisted of a 10-day baseline monitoring period followed by a 30-day intervention period. Prior to the start of the intervention, author AJR randomized participants into 2 groups in order of the date they enrolled into the study, using an online random number generator. Groups were defined as follows: the experimental group (APP), who had access to the AI dietary mobile app and standard dietary education materials, and the control group (CON), who only had access to standard dietary education materials. Researchers and participants were both unblinded to the intervention type. Both groups received standard online LFD intervention resources developed by the University of Michigan [31]. The online education provided a description of the LFD and instructions for adherence, a high and low FODMAP guide, and an LFD cooking guide that contained 21 sample meals. The APP group received the same educational materials in addition to access to the Heali mobile app. Standardized emails were sent out to the APP and CON groups, which provided resources and questionnaires to support and track participation. Upon completion of the study, participants were also provided a guide for the reintroduction of FODMAPs, the final stage of the low FODMAP diet, which they were able to tailor to their needs. Those who successfully completed the intervention were able to use the AI mobile application to support their reintroduction stage over a 6-month period after completion.

### Heali Mobile App

For this study, APP participants only had access to the LFD, meaning they had access to all features within the app with the exception of the ability to further personalize their preferences beyond the LFD. They also had access to a within-app health coach, which provided participants weekly reminders to use the app and answered questions elicited by the participants. APP participants were instructed to use the app daily. Participants gained access to the app via a cell phone login and received a standard usage tutorial in PDF form.

### Measures

Prior to the 10-day baseline period and during the screening process, participants completed 3 questionnaires, including a demographic screener (inclusion criteria and cofounding variables, such as physical activity [32], smoking, and history of disease), the IBS-SSS diagnostic tool [33], and the Rome IV screener [29]. All questionnaires were sent via Google Forms and deployed in an email to participants. Once accepted into the study, all participants were required to complete 5 questionnaires at the start of the 30-day intervention period and at the end of the trial, specifically the Rome IV questionnaire, the IBS-SSS diagnosis tool, the low FODMAP dietary consumption (LFDA) questionnaire, the low FODMAP dietary knowledge (LFDK) questionnaire, and a quality of life questionnaire. The 6-item Rome IV questionnaire (1-2 minutes to complete) is the current standard diagnostic tool developed by the Rome Foundation used to determine severity of GI dysfunction and diagnose IBS [29]. The 5-item IBS-SSS questionnaire (1-2 minutes to complete) is a 500-point symptom severity screener validated by Francis et al [33]. Respondents were categorized into 1 of 4 categories based on their responses: <75, no symptoms; 75 to <175, mild IBS; 175 to <300, moderate IBS; and ≥300, severe IBS [33]. The IBS-SSS was also completed once every 10 days over the course of the trial. The 110-item LFDA questionnaire (based on the NHANES food frequency questionnaire) recorded the number of times daily that respondents ate certain FODMAP items [34]. The total scoring range was 0-385, with 0 indicating no FODMAPs eaten in the last month and 385 indicating every FODMAP was eaten 2-3 times per day or more [34]. The 12-item LFDK questionnaire quantified the respondents’ knowledge of LFD. The total scoring range was 0-60, with 60 being the best possible knowledge score. The survey was modified from a 12-item validated tool by Krause et al [35] to assess FODMAP knowledge rather than general nutrition knowledge. These modifications included the following: “When I have questions on FODMAP foods, I know where I can find information on this issue,” where “FODMAP foods” was substituted for “healthy nutrition” for the purposes of this study. The 13-item quality of life measure represented domains 1 and 2 of the World Health Organization (WHO) quality of life questionnaire, in which the total scoring range was 0-200, with 200 being the best possible quality of life score [36].

### Statistical Analysis

Data are presented as mean (SD). Analyses were conducted using SPSS Statistics Version 26 (IBM Corp) for Windows (Microsoft Corp). Spearman test was used to determine correlations in baseline variables. Mann-Whitney U tests were used to determine differences between groups. Pearson chi-square test was used to determine differences for nominal data. All tests used were 2 sided, and a *P* value <.05 was considered significant.

## Results

### Participants

**Figure 1:**
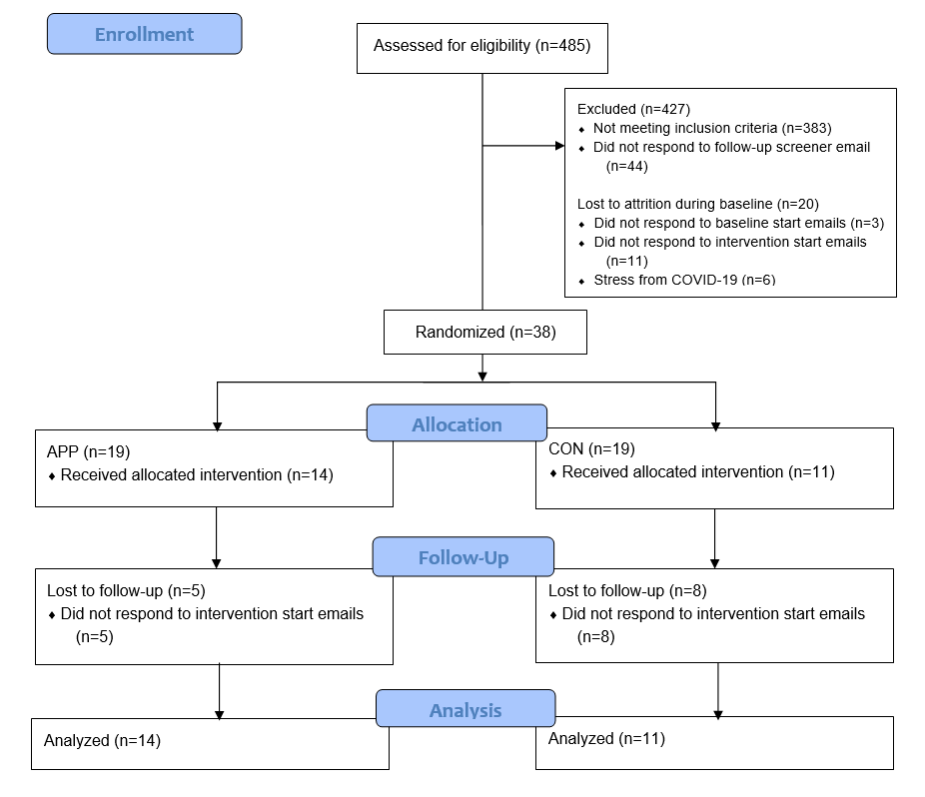
CONSORT (Consolidated Standards of Reporting Trials) flow diagram. APP: experimental group (access to artificial intelligence dietary mobile app); CON: control group.

We enrolled 58 adults who met the study criteria, 20 participants were lost to follow-up during the 10-day baseline period, and 38 participants were randomized to the treatment arm (APP, n=19) and control arm (CON, n=19; Figure 1).

During the intervention period, 13 participants were lost to attrition, and 25 participants completed the study in its totality (CON, n=11; APP, n=14). The COVID-19 pandemic accelerated during the intervention period and accounts for some of the attrition, as stated by those participants who responded to feedback questionnaires. Baseline characteristics at the start of the intervention phase for those completing the study did not differ significantly between the CON and APP groups (Table 1).

**Table 1:** Baseline characteristics.

Participant characteristics		CON^a^ (n=11)	APP^b^ (n=14)
**Gender, n**			
	Male	1	2
	Female	10	12
Age (years), mean (SD)		25.7 (11.9)	27.2 (9.5)
BMI (kg/m^2^), mean (SD)		25.0 (3.3)	27.7 (5.8)
Physical activity (METS/wk^c^), mean (SD)		57.8 (39.3)	60.1 (37.0)
**Rome IV, n**			
	Constipation	4	1
	Diarrhea	3	8
	Mixed	4	5
IBS^d^ symptom score, mean (SD)		275 (56)	272 (43)
Low FODMAP^e^ knowledge score, mean (SD)		32.6 (6.5)	29.8 (8.5)
Low FODMAP adherence score, mean (SD)		57.3 (21.8)	65.6 (26.6)
Quality of life score, mean (SD)		117.2 (32.5)	107.4 (27.4)

^a^CON: control group.

^b^APP: experimental group (access to artificial intelligence dietary mobile app).

^c^METS/wk: metabolic equivalents per week.

^d^IBS: irritable bowel syndrome.

^e^FODMAP: fermentable, oligo-, di-, monosaccharide and polyol (diet).

### IBS Diagnosis

IBS diagnosis, as measured by the Rome IV criteria, decreased in both groups after the intervention. In the APP group, 6 of 14 participants no longer met the criteria for IBS diagnosis after completion of the intervention. In the CON group, 5 of 11 participants no longer met the criteria for IBS diagnosis at the end of the trial. IBS-D was the most common diagnosis at study completion (CON: IBS-D=2; APP: IBS-D=5); IBS-C and IBS-M were the least diagnosed conditions at study completion (CON: IBS-C=2, IBS-M=2; APP: IBS-C=2, IBS-M=1).

### IBS-SSS

**Figure 2:**
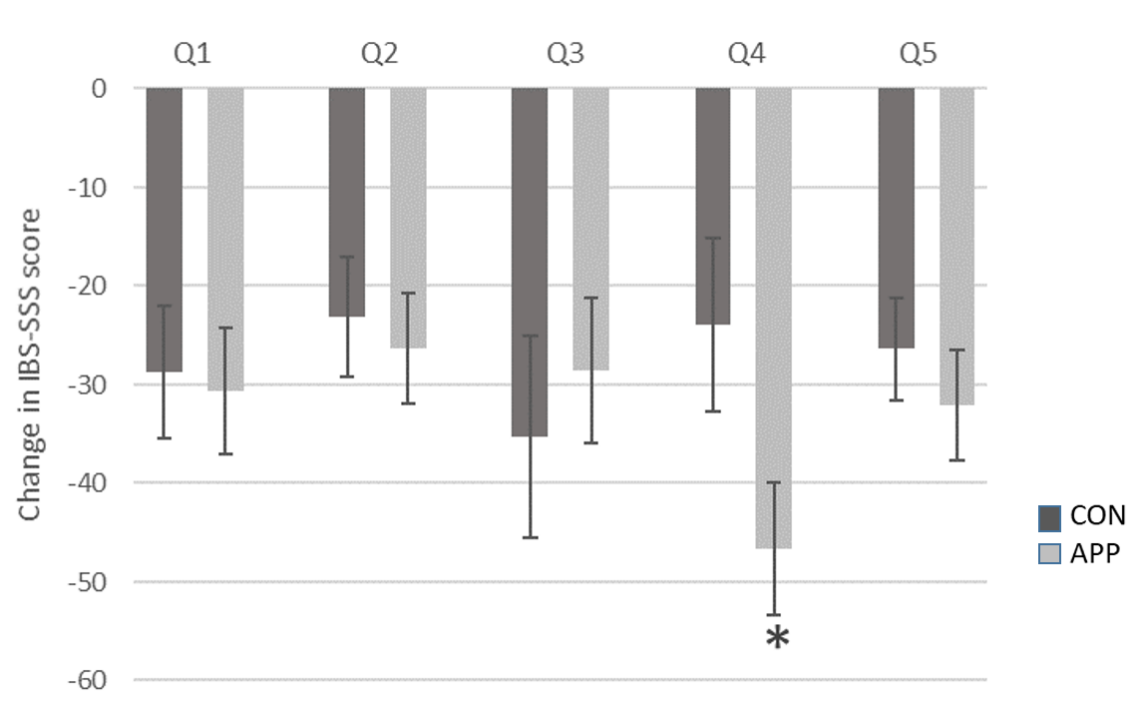
Thirty-day change (mean±SD) in IBS-SSS scores by question. APP: experimental group (access to artificial intelligence dietary mobile app); CON: control group; IBS-SSS: irritable bowel syndrome symptom severity scale; Q1: abdominal pain severity; Q2: abdominal pain frequency; Q3: abdominal pain distension severity; Q4: bowel habit dissatisfaction; Q5: quality of life interference due to the aforementioned symptoms. *Significant difference between CON and APP groups (*P*=.05, Mann-Whitney U test).

The total IBS symptom scores improved over the intervention period for the sample as a whole; however, the change in scores did not differ significantly between groups (CON: –138; APP: –170; *P*=.37; Table 2). Of the 5 individual symptom component scores, the bowel habit dissatisfaction score showed a significant between-group reduction in symptoms. The APP group reported a 2-fold improvement in bowel habit scores in comparison to the CON group (APP: –47; CON: –24; *P*=.05; Figure 2). Significant improvements were noted for the remaining 4 component scores over the 30-day intervention within both groups, but these changes did not vary significantly between groups.

**Table 2:** Thirty-day change in survey scores.

Survey	CON^a^ (n=11), median (IQR)	APP^b^ (n=14), median (IQR)	*P* value^c^
IBS^d^ symptoms	–123 (–235, –57)	–165 (–238, –116)	.37
Low FODMAP^e^ knowledge	10.4 (7.4, 14.0)	8.3 (4.4, 13.1)	.50
FODMAP intake	–6.0 (–27.0, 9.5)	–14.0 (–41.4, 3.3)	.85
Quality of life	7.0 (6.0, 18.0)	21.5 (12.0, 40.3)	.04

^a^CON: control group.

^b^APP: experimental group (access to artificial intelligence dietary mobile app).

^c^*P* values were determined by Mann-Whitney U test.

^d^IBS: irritable bowel syndrome.

^e^FODMAP: fermentable, oligo-, di-, monosaccharide and polyol (diet).

### Low FODMAP Diet Intake Knowledge

LFD knowledge scores improved over time in both the APP and CON groups, but there was no difference between groups for change in knowledge scores. Adherence to the LFD improved during the study as indicated by a decrease in FODMAP intake scores over the intervention period for both groups. However, the change in intake scores between groups following the intervention period did not differ significantly (Table 2).

### Quality of Life

Quality of life scores improved to a greater degree in APP participants compared to CON participants (*P*=.04; Table 2). Furthermore, improvement in quality of life scores was correlated to improvement in IBS symptom scores in the APP group (*r*=–0.598; *P*=.02) but not in the CON group (*r*=–0.183; *P*=.59).

## Discussion

### Principal Results

These data suggest that supplementing standard IBS dietary education with an AI dietary mobile app that tailors the LFD to specific users’ needs improves several health outcomes for individuals with IBS. Specifically, the Heali mobile app helped participants improve their quality of life outcomes and bowel habit symptoms. Quality of life scores rose in both groups; however, the rise in the APP group was 2.6-fold greater than that in the CON group. Poor quality of life is well documented in patients with IBS, and improvement in IBS symptoms is related to improvement in quality of life, such as decreasing the cost of health care, fewer missed days of work, and greater sense of control [37].

### Comparison With Prior Work

Similar to the present trial, Kortlever et al [38] recently demonstrated that adoption of the LFD in IBS patients improved GI symptoms and quality of life after 6 weeks of diet adherence. To achieve these results, patients consulted with dietitians at private dietary centers for detailed diet reviews and personalized diet counseling. The data herein suggest that similar outcomes can be achieved with a mobile app, which can thus eliminate certain barriers, such as time commitment, cost, and access to health professionals, making IBS treatment accessible and convenient for a large segment of the population. Although significant quality of life improvements were found in the app group, Pederson et al [10] found that IBS-D participants have a greater response to web-based treatment specifically for quality of life outcomes. In this study, IBS-D participants made up 57% of those in the APP group and 23% of those in the CON group. Although this study was a pilot, future iterations should stratify by IBS subtype to evenly spread this confounding factor.

It is noteworthy that both treatments presented herein improved outcome measures, including diet adherence; yet, mobile app use did demonstrate added benefits. Consumers are increasingly relying on their smartphones for news and information and to conduct their personal business, including managing their health. Mobile apps that provide ready access to accurate, detailed, and personalized diet information can enable individuals to make lifestyle changes with confidence to improve health. Heali provides evidence-based information and real-time feedback on food choices via a personalized match rating (using the traffic light system) to support user adherence to difficult diets like the LFD.

To our knowledge, Heali is the first mobile app to use AI to support dietary adherence to the LFD and to support treatment of IBS symptoms. This is significant considering that peer-reviewed mobile apps using AI are able to optimize support of chronic disease treatment of conditions such as diabetes [23,39] and hypertension [25]; however, these apps have not been tested on less prominent conditions like IBS. This app-based intervention is especially novel as few studies have explored the possibilities of AI to improve dietary adherence, especially to the LFD [23,26,39]. This cements the novelty of this study on two fronts: (1) the use of AI to improve quality of life in patients with IBS and (2) the use of AI to improve IBS disease outcomes.

### Strengths and Limitations

The strengths of this study include the randomization of participants to the intervention and control groups, which allowed dispersion of confounding variables between groups. Further, the entire study, from recruitment to implementation and assessment, was completed online, allowing broad participation without location limitations. The online nature of the study also decreased study costs, as it only required desktop support for implementation and completion.

The limitations of this study include the potential for self-reporting bias, as all surveys were conducted online, while there was also a lack of blinding of the researcher and the participant to the study groups. Participants were randomized via random number generation and not stratified based on severity or type, presenting the potential for bias when comparing between groups. However, participants were not randomized to groups until study day 10, which was after the collection of baseline data. Although anthropometric measures (bodyweight, height) were collected at baseline, they were not collected at the end of the trial, and it is possible that changes in body weight influenced the outcome variables. The change in quality of life was not related to baseline bodyweight or BMI. The small sample size of this pilot trial limits the ability to interpret or generalize findings to other patient populations. Finally, all participants completed the majority of their intervention over the early course of the 2020 COVID-19 pandemic, which reportedly affected participation and retention. Given that this was a dietary restriction study in participants with IBS, it is important to note that household goods (eg, nonperishable foods, proteins, and paper products such as toilet paper and paper towels) were in scarce supply during this time. As a result, it is likely that COVID-19 affected adherence across both groups potentially even more than was stated by participants. The COVID-19 pandemic likely also led to elevated stress, which, in addition to dietary nonadherence, has been shown to exacerbate IBS symptoms [7,8,40,41]. It is therefore important to consider that results reported on symptom severity screeners, progression of the disease, and the quality of life of the participants within this study might have been negatively impacted by the COVID-19 outbreak.

### Conclusions

This pilot study provides preliminary evidence that the Heali app may provide therapeutic benefit to its users with IBS. Results showed that the Heali app was able to significantly increase quality of life outcomes in IBS participants over a 30-day intervention period. These findings warrant further research using larger sample sizes. Although this study focused on patients with IBS and the LFD, the variety of additional interventions available via the Heali app suggest possible benefits to individuals with other conditions whose symptoms are attenuated through therapeutic dietary adherence.

## Appendix

Multimedia Appendix 1 CONSORT-eHEALTH checklist (V 1.6.1).

## Abbreviations

AI: artificial intelligence
APP: experimental group (access to artificial intelligence dietary mobile app)
CON: control group
FODMAPs: fermentable, oligo-, di-, monosaccharides and polyols
GI: gastrointestinal
IBS: irritable bowel syndrome
IBS-C: IBS with constipation
IBS-D: IBS with diarrhea
IBS-M: IBS mixed typed
IBS-SSS: IBS symptom severity scale
IBS-U: IBS unsubtyped
LFD: low FODMAP diet
LFDA: low FODMAP dietary consumptionˍ
LFDK: low FODMAP dietary knowledge
WHO: World Health Organization
